# A Standards Organization for Open and FAIR Neuroscience: the International Neuroinformatics Coordinating Facility

**DOI:** 10.1007/s12021-020-09509-0

**Published:** 2021-01-27

**Authors:** Mathew Birdsall Abrams, Jan G. Bjaalie, Samir Das, Gary F. Egan, Satrajit S. Ghosh, Wojtek J. Goscinski, Jeffrey S. Grethe, Jeanette Hellgren Kotaleski, Eric Tatt Wei Ho, David N. Kennedy, Linda J. Lanyon, Trygve B. Leergaard, Helen S. Mayberg, Luciano Milanesi, Roman Mouček, J. B. Poline, Prasun K. Roy, Stephen C. Strother, Tong Boon Tang, Paul Tiesinga, Thomas Wachtler, Daniel K. Wójcik, Maryann E. Martone

**Affiliations:** 1 INCF Secretariat, Karolinska Institutet, Stockholm, Sweden; 2 Institute of Basic Medical Sciences, University of Oslo, Oslo, Norway; 3 McGill Centre for Integrative Neuroscience, McGill University, Montreal, QC, Canada; 4 Monash Biomedical Imaging, Monash University, Clayton, VIC, Australia; 5 McGovern Institute for Brain Research, Massachusetts Institute of Technology, Cambridge, MA, USA; 6 Department of Otolaryngology - Head and Neck Surgery Harvard Medical School Boston, Boston, MA, USA; 7 Monash eResearch Centre, Monash University, Melbourne, VIC, Australia; 8 Department of Neuroscience, School of Medicine, University of California, San Diego, La Jolla, CA, USA; 9 KTH Royal Institute of Technology, School of Electrical Engineering and Computer Science, Stockholm, Sweden; 10 Centre for Intelligent Signal and Imaging Research, Institute of Health and Analytics, Universiti Teknologi PETRONAS, Perak, Malaysia; 11 Department of Psychiatry, University of Massachusetts Medical School, Worchester, MA, USA; 12 Serendipitea.World, Hasselby, Sweden; 13 Nash Family Center for Advanced Circuit Therapeutics, Icahn School of Medicine, New York, NY, USA; 14 Institute of Biomedical Technologies, National Research Council (CNR), Milan, Italy; 15 Department of Computer Science and Engineering, Faculty of Applied Sciences, University of West Bohemia, Pilsen, Czech Republic; 16 Montreal Neurological Institute, Faculty of Medicine and Health Sciences, McGill University, Montreal, Canada; 17 Computational Neuroscience & Neuroimaging Laboratory, School of Bio-Medical Engineering, Indian Institute of Technology (BHU), Varanasi, UP, India; 18 Rotman Research Institute, Baycrest Centre, Department of Medical Biophysics, University of Toronto, Ontario, ON, Canada; 19 Centre for Intelligent Signal and Imaging Research, Institute of Health and Analytics, Universiti Teknologi PETRONAS, Bandar Seri Iskandar, Malaysia; 20 Donders Institute for Brain, Cognition and Behaviour, Radboud University Nijmegen, Nijmegen, Netherlands; 21 Department of Biology II, Ludwig-Maximilians-Universität München, Martinsried, Planegg, Germany; 22 Laboratory of Neuroinformatics, Nencki Institute of Experimental Biology of Polish Academy of Sciences, Warsaw, Poland

**Keywords:** Neuroinformatics, Standards and best practices, FAIR principles, Standards organization, Neuroscience, INCF, INCF endorsement process

## Abstract

There is great need for coordination around standards and best practices in neuroscience to support efforts to make neuroscience a data-centric discipline. Major brain initiatives launched around the world are poised to generate huge stores of neuroscience data. At the same time, neuroscience, like many domains in biomedicine, is confronting the issues of transparency, rigor, and reproducibility. Widely used, validated standards and best practices are key to addressing the challenges in both big and small data science, as they are essential for integrating diverse data and for developing a robust, effective, and sustainable infrastructure to support open and reproducible neuroscience. However, developing community standards and gaining their adoption is difficult. The current landscape is characterized both by a lack of robust, validated standards and a plethora of overlapping, underdeveloped, untested and underutilized standards and best practices. The International Neuroinformatics Coordinating Facility (INCF), an independent organization dedicated to promoting data sharing through the coordination of infrastructure and standards, has recently implemented a formal procedure for evaluating and endorsing community standards and best practices in support of the FAIR principles. By formally serving as a standards organization dedicated to open and FAIR neuroscience, INCF helps evaluate, promulgate, and coordinate standards and best practices across neuroscience. Here, we provide an overview of the process and discuss how neuroscience can benefit from having a dedicated standards body.

## Introduction

With major brain initiatives across Asia, North America, and Europe committing significant resources to large-scale, multifaceted efforts to understand the nervous system, we are likely entering a golden age for neuroscience. At the same time, neuroscience, like many domains in biomedicine, is undergoing a reproducibility crisis, where small, underpowered studies, problems in experimental design and analysis, and lack of routine data sharing lead to difficulty in relying on published results (Button et al. 2013).

Common to both the large brain projects and individual investigator led research is the recognition that neuroscience as a whole needs to converge towards a more open and collaborative enterprise with neuroscientists around the globe committed to open sharing of data and tools. The Declaration of Intent of the International Brain Initiative,^1^ an alliance of large national brain projects, states: “Researchers working on brain initiatives from around the world recognise that they are engaged in an effort so large and complex that even with the unprecedented efforts and resources from public and private enterprise, no single initiative will be able to tackle the challenge to fully understand the brain”.

Effective resource sharing means not just that data, processing methods, workflows, and tools are made available, but that they can be discovered and are made available in a way that ensures that published findings can be reproduced. Currently, it has been estimated that over 80% of the time spent in handling data goes not to the analysis, but to data preparation: 60% of time for cleaning and organizing data and 19% of time spent collecting datasets (Gil Press 2016); and curation for dataset integration requires more resources than generation of the data (Palsson and Zengler 2010). Of equal importance, in the age of machine learning and artificial intelligence, data should be published with integration and reuse in mind, so they can be interpreted in new ways and leveraged so that new knowledge can be extracted (Ferguson et al. 2014). For that to happen, neuroscience as a discipline needs to adopt the FAIR principles (Wilkinson et al. 2016), ensuring that the results of science are Findable, Accessible, Interoperable and Reusable, to both humans *and* machines. FAIR neuroscience means that neuroscientists world-wide, working in big team projects or individual laboratories acquire, manage, and share digital resources so that they can be reliably compared, aggregated, and reused. As neuroscience becomes a FAIR discipline, the grand challenge of piecing together a more comprehensive understanding of nervous system structure and function from multiple data sets should become more feasible.

The FAIR principles were formulated in a collective effort by several international groups, based on practical experience of the roadblocks encountered when trying to reuse data, particularly public data. The high level principles are summarised into a set of 15 attributes that represent best practices for FAIR. Some recommendations are domain independent, e.g., proper licenses, use of persistent identifiers. Other recommendations, however, particularly those that address interoperability and reusability, delegate the specifics to individual scientific communities, who are required to define the relevant standards and best practices for their specialized data types and protocols. So how does neuroscience with its vast number of subdisciplines, techniques, data types, and model systems become a FAIR discipline?

First, FAIR requires that the necessary infrastructure in the form of web-accessible repositories is available to neuroscientists for publishing research objects: data, code, and workflows. These repositories should support FAIR and implement basics such as persistent identifiers, programmatic access, and clear licenses. Second, neuroscience needs the means to define and support “community-relevant” standards both for data and metadata. Such standards include common formats (e.g., NifTI; (Cox et al. 2004), file structures (e.g., BIDs, (Gorgolewski et al. 2016)), data elements (Sheehan et al. 2016), markup languages (e.g., odML, NeuroML, NineML (Grewe et al. 2011); (Cannon et al. 2014); (Raikov et al. 2014)) metadata standards such as minimal information models (e.g., COBIDAS, (Nichols et al. 2017)), protocols and machine-readable “FAIR” vocabularies (e.g., NIFSTD ontology, (Bug et al. 2008). For neuroscience, with its diverse data types, dynamics and scales, such standards need to include the necessary information for understanding what areas of the nervous system were studied and from which structures data were acquired under which conditions.

As in many disciplines, standards in neuroscience have been developed on an “as needed” basis with many different starting points. For instance, the Connectivity File Formats Documentation (cifti) format was developed internally in the Human Connectome Project as a standard for storing both surface and volumetric imaging data, tailored to the specific needs of the project. The Neuroimaging Informatics Technology Initiative (Nifti) image format was developed under the umbrella of the US National Institutes of Health (NIH) which acted as a broker. Adoption of the format was ensured by involving developers of all the major brain imaging analysis tools and their commitment to implement the standard. Similarly, a joint effort by neurophysiology data acquisition systems vendors to define a common format led to the neuroshare standard (neuroshare.org); while being seen as far from ideal, cifti, Nifti, and the neuroshare standard have been in wide use by the community and undoubtedly have enabled re-use of data to an extent that otherwise would not have been possible.

Beyond clinical standards such as FHIR,^2^ convergence on disease-specific standards for data collection, Common Data Elements (CDEs^3^), is resulting in some early successes where data collected across different centers and even countries is comparable. For example, a cross-European study of traumatic brain injury, CENTER-TBI^4^ has used CDEs and other data collection standards to integrate data from 21 European countries and 3 countries beyond Europe (Maas et al. 2017). However, harmonizing CDEs and other clinical data standards across broader international boundaries remains a challenge, although recent progress has been made in the form of the guidelines for Data Acquisition, Quality, and Curation for Observational Research Designs (DAQCORD; Ercole et al. 2020).

Issues in the development and use of standards fall into several broad technical and sociological categories. At the forefront is the paradoxical nature of the standards landscape where the availability of too many overlapping standards leads to too few being adopted, as a well known cartoon illustrates.^5^ It is common in scientific domains, where researchers are generally rewarded for novelty, that research funding ends up producing multiple potential standards, many of which lack the required documentation, tooling, or community support for wide adoption and long term sustainability. As an example in genomics, FAIRsharing.org^6^, a database that keeps track of standards for biomedical science, lists 38 standards for “gene expression data” of which 24 have a publication associated. Seventeen of these have a maintainer listed, but only three are recommended (by Biomed central, EMBO, Giga Science, or Scientific data). Only one has all three: publications, a maintainer, and evidence of use.

The overhead of having to account for multiple standards in neuroscience research is very high. With multiple competing standards, those developing tools may need to implement and maintain several input/output interfaces or develop format conversion routines, draining time and money away from more critical tasks. For example, Neo, a Python package for representing electrophysiology data, provides IO modules for ~20 different electrophysiology formats.^7^ With poorly documented or out of date standards, projects may invest in a standard to accommodate immediate needs, only to find that it hasn’t achieved widespread uptake and therefore outputs are not FAIR.

In areas that benefit from well documented and validated standards, standards organizations or standards bodies play a central role in the adoption and promotion of standards and best practices. Standards organizations like the W3C and IEEE have as their primary activity the development, coordination, promulgation, and upkeep of technical standards that are intended to address the needs of a group of affected adopters (e.g., Web browser developers, hardware developers; (Wikipedia contributors 2018b). They establish criteria by which standards and best practices can be evaluated and a means for community vetting to ensure that the standard is needed and functions appropriately. Such criteria include the availability of proper validation tools and implementations.

Standards efforts in basic science are also propelled by dedicated organizations such as the Research Data Alliance (rd-alliance.org) to provide a substrate whereby communities can come together to define a needed standard, or to provide coordination among different standards’ efforts to ensure interoperation. For example, the Computational Modeling in Biology Network (COMBINE^8^), is an initiative composed of those developing standards and tools for computational modeling, whose goal is to “coordinate the development of the various community standards and formats for computational models. By doing so, it is expected that the federated projects will develop a set of interoperable and non-overlapping standards covering all aspects of modeling in biology.”

Neuroscience, whether basic, clinical or computational, similarly will benefit from having a dedicated standards organization to help support the ambitious goals of international brain projects and the needs of individual investigators, including the necessity to formally publish data and tools in an effective manner. The International Neuroinformatics Coordinating Facility (INCF) has been actively working in the area of standards and infrastructure for neuroscience over the past decade. Here, we outline how INCF is evolving its operations to promote open and FAIR neuroscience across international boundaries. In particular, INCF is taking on a more formal role as a standards organization for neuroscience, by extending their work in standards to include the evaluation, coordination, and endorsement of community standards. Through this process, neuroscientists and big brain projects will have uniform, unbiased and independent analysis of neuroscience standards and best practices, to ensure that standards are robust, well supported and documented.

### INCF as a Standards Organization

The International Neuroinformatics Coordinating Facility (INCF) was launched in 2005 as an independent international organization dedicated to promoting the sharing of neuroscience data, data reuse and reproducibility, through the coordination of infrastructures and standards. Based on recommendations from the Organisation for Economic Co-operation and Development (OECD), an international agency of over 30 countries comprising the world’s leading economies, the INCF instituted a national membership model, whereby individual nations establish a national neuroinformatics Node and is represented in INCF governance structures. Since 2016, the governance framework has consisted of the Governing Board, comprising national-level funding representation from those Nodes that financially sustain the organisation (Governing Nodes), and an additional Council for Training, Science and Infrastructure (CTSI) which comprises scientific and infrastructural representation from all INCF Nodes (Governing and Associate Nodes), as well additional appointed international experts. The CTSI recommends INCF’s scientific, infrastructural and training direction and appoints specialist subcommittees such as Training & Education, Infrastructure, Standards and Best Practices, and FAIR. A Secretariat based at the Karolinska Institute in Sweden manages the coordination operations of the organization.

From 2007 to 2016, INCF operated scientific programs on topics requiring coordination and cooperation across national boundaries. Community needs and requirements were defined through topical international scientific workshops.^9^ Building on these identified areas, the Governing Board instantiated a steering committee comprising international experts in the field to have oversight of each scientific program. Working with the Secretariat, the steering committee initiated actions (top-down) which included launching one or more task forces to address the issues, develop technical specifications, make recommendations and develop appropriate tools or infrastructure. The INCF task forces each operated for a few years to deliver these technical solutions, many outreaching also to the broader international community.

Under this model, the INCF yielded a number of successes, e.g., the Waxholm space atlas interoperability framework ((Johnson et al. 2010); (Hawrylycz et al. 2011); (Papp et al. 2014)), the neuroimaging data model: NIDM (Sochat and Nichols 2016) and others listed in Table 1. In these initial efforts and early days in neuroinformatics, the INCF focused most heavily on de novo development of standards, serving as a broker for standards development across stakeholder groups.

However, this earlier INCF model for standards development was subject to limitations and criticisms. The process was expensive to maintain and often too slow to keep pace with the launch of new projects or development of new technologies. It lacked a formal means for evaluation of resulting standards and for community input into the process. Also, it had no formal mechanism for promoting and encouraging the use of already existing standards and best practices, nor a formal governance procedure to help adjudicate among competing interests.

The INCF has undergone a significant reorganization over the past 4 years to allow it to be more responsive to the needs of the global neuroscience community and more transparent in its operations. Rather than a top down governance model where a steering committee sets priorities, INCF adopted successful models from other community organizations like FORCE11 (www.force11.org) and the Research Data Alliance (RDA; www.rd-alliance.org/) to increase community participation and a sense of ownership over the process. INCF has launched a new system of community-driven scientific interest groups, where groups of neuroscientists can come together to work on an issue of particular interest in the area of neuroinformatics. Oversight and guidance is provided by the CTSI with its international scientific representation from INCF member Nodes and external expertise.

As part of this reorganization, INCF has developed a formal and community-focused process whereby standards are considered and endorsed. The process includes a pathway for both community nomination and committee invited submissions of SBPs spanning data collection to publication, evaluation against a consistent set of criteria, and active solicitation of community feedback. An important change for INCF is that these standards and best practices need not have been developed by INCF sanctioned groups or even be specific to neuroscience. Indeed, one of the goals is to ensure that neuroscience can benefit from the work that has gone on in other biomedical or scientific domains around FAIR data. For example, INCF may choose to endorse standards such as the ORCID, the unique identifier for researchers, or the FAIR principles themselves. In this way, INCF can promote initiatives emerging in different scientific domains that bring neuroscience data into alignment with widely accepted standards and principles. This approach also allows INCF to fulfill its coordinating role by offering sets of endorsed practices, and to select, prioritize, and possibly stimulate further development and convergence of overlapping standards. As an independent organization with broad international reach and neuroinformatics expertise, INCF is uniquely positioned and experienced to act as a standards endorsing authority for neuroscience.

### The INCF Standards and Best Practices Endorsement Process

Through a series of community meetings and interactions with representatives from national standards organizations like the Standard and Industrial Research Institute of Malaysia (SIRIM) and the US National Information Standards Organization (NISO), the CTSI developed a set of criteria and an initial process for evaluating standards and best practices (SBPs) against criteria that support open and FAIR neuroscience (Table 2). The term “best practices” was added in recognition that many of the requirements for open and FAIR neuroscience may not involve an actual technical standard, such as a file format. Rather best practices involve practices that are accepted as producing better results than those achieved by other means (Wikipedia contributors 2018a), and that should become standard operating procedure for experimental neuroscience, e.g., making sure that researchers reference their data to a standard brain atlas when reporting on location.

A call went out in spring of 2018 for nominations of SBPs from the community and a standing committee was formed to establish the necessary procedures and infrastructure for review and voting. The SBP Committee operates under the auspices of the CTSI and is composed of a representative from each of the INCF Governing Nodes, and members from two of the Associate Nodes (currently the US and Germany). Since 2019, a more formal procedure for committee membership has been implemented to ensure broad community participation in the process.

As a first step, the SBP committee established a more detailed set of criteria for evaluation based on seven key areas:

1. **Open:** Is the SBP open according to the Open Definition^10^ and does it follow open development practices?
2. **FAIR:** Considers the SBP from the point of view of relevant FAIR criteria (Wilkinson et al. 2016). Is the SBP itself FAIR? Does it result in the production of FAIR research objects? Some of these criteria may not apply in all cases.
3. **Testing and implementation:** Is the SBP supported by appropriate software, that is open, well designed, implemented, validated, documented and available for use?
4. **Governance:** Does the SBP have a governance structure that makes it clear how decisions are made and how grievances are handled?
5. **Adoption and use:** The SBP must have substantive evidence of use outside of the group or individual that develops and maintains it. Because INCF is an international organization, evidence of international use is a requirement.
6. **Stability and support:** Who is actively maintaining and supporting the SBP and what are the plans for long term sustainability?
7. **Comparison with other SBP’s:** Competing standards add extra burden to the community. The INCF seeks to endorse only a single standard per area, unless the suggested approach is complementary as further discussed below.

Under each of these areas, a set of questions were developed to aid reviewers in evaluating how well an SBP complied with each criteria. Version 1 of the review criteria (Standards and Best Practices Committee 2019a) are shown in Table 2.

**Fig. 1:**
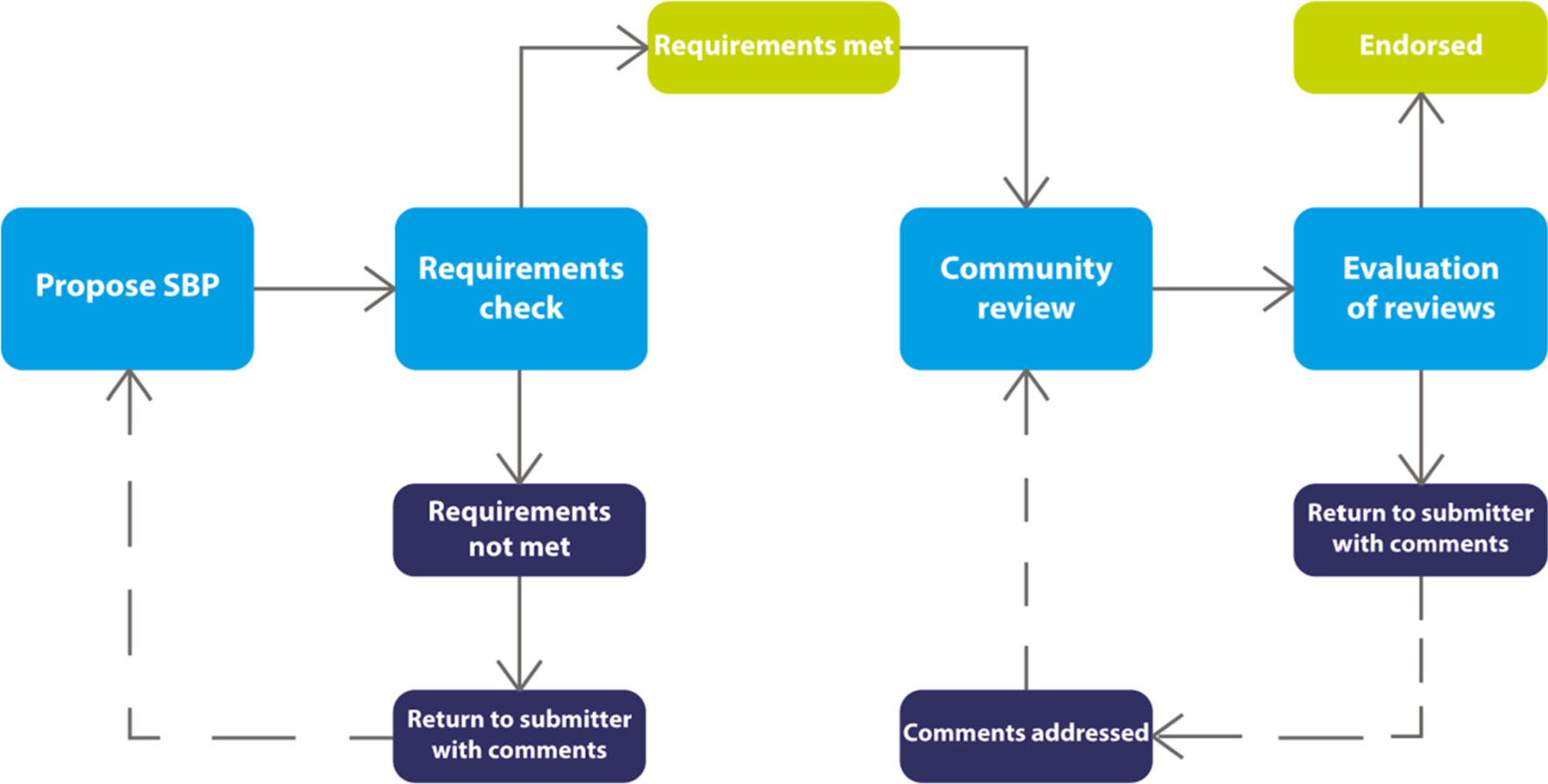
A schematic representation of the INCF SBP submission, review and endorsement process

Once the criteria were established, the committee developed a basic procedure for the evaluation, starting with community nomination or in response to an invitation from the committee to submit an SBP. From the first SBP nominations, BIDS (the Brain Imaging Data Structure; http://bids.org), a standard for organizing and naming files generated during a neuroimaging experiment, was chosen as the initial test case. The current procedure is shown schematically in Fig. 1 and comprises the following steps:

1. SBP is received by the INCF through an on-line submission form. SBP submissions are received as the result of direct submission, in response to a broad call for submissions, or in response to direct invitation from the committee.
2. If the SBP is determined to be in scope, the developer/steward of the SBP is contacted and asked to provide some details about the SBP according to the criteria outlined in Table 2.
3. The Committee assigns 2–3 reviewers, committee members or external experts, to review the materials and conduct an independent analysis. Reviewers should have no conflicts of interest that would preclude an impartial analysis of the SBP.
4. After initial review, the full committee votes on whether to accept the SBP for consideration or to reject it.
5. If accepted, a write up of the SBP is prepared and posted for community input. For BIDS, the text was posted on the INCF’s F1000 channel (Martone et al. 2018) and on Google Docs.
6. Feedback is solicited through announcements via the INCF and the Node Network’s social and media channels. The comment period is 60 days from posting.
7. After the commenting period, the reviewers review the feedback and decide whether the comments require further review.
8. Once the review is complete, the committee votes on whether to endorse the SBP.
9. If endorsed, the stewards/authors are allowed to display the “Endorsed by INCF logo” on their website.
10. Endorsed standards are displayed on the INCF website and actively promulgated through INCF training activities.
11. Endorsed standards are re-evaluated every 2 years to ensure that they are still relevant or need to be replaced.

As of this writing, INCF has completed the reviews of 6 standards, endorsed 5, and is in the process of reviewing an additional 2 submitted standards (Table 3). We are using this initial round of submissions to develop and test the review process, including both the criteria used and the governance of the process itself, e.g., how does the SBP committee handle conflicts of interests within the committee.

INCF is also developing additional materials and tools to help the neuroscience community identify and use appropriate standards, e.g., a catalog to navigate and assess relevance of endorsed SBP’s for their work, and training materials and workshops designed to guide neuroscientists and tool developers in their use. To fulfill its coordinating role, those working on SBP’s ranging from data collection to publication can request support to form a working group to develop a standard in an area in need of standardization and address issues such as extension of endorsed standards to cover different domains and harmonization of existing standards. INCF actively solicits input from the community on areas in neuroscience in need of standardization through its thematic workshops and a submission form on the INCF website where community members can recommend an area in neuroscience in need of standardization (e.g. methods standardization) whether they are willing to work on it or not; under this framework, INCF hosts thematic workshops to determine requirements and supports working groups to develop to the SBP. Any work performed by INCF-supported groups will be subjected to the same type of rigorous review as outside SBP’s to achieve INCF endorsement. We expect the INCF endorsement process to further evolve over time to confront the challenges inherent in a dynamic and distributed research landscape. Some of the known challenges involve establishing open and transparent governance for the endorsement process that recognizes and seeks to balance the competing needs of different stakeholder groups. Another key issue is the extension and evolution of SBPs over time.

### Governance

The INCF SBP committee operates in a transparent manner and seeks to avoid at all times any type of bias or appearance of bias. The process should be fair to those who are developing SBP’s, but also in the best interests of the broader neuroscience community that we seek to serve. Although the process is still being refined, it was designed to be open, collegial, and transparent. Reviewers are not anonymous and are required to clearly state whether they have a conflict of interest. Committee members with conflicts do not participate in the reviewing or voting process. At each step—preparation of review documents, posting of the review for community feedback, and post-feedback synthesis—reviewers are encouraged to contact the SBP provider for additional information and to provide feedback on issues that might be addressable, e.g., indicating a clear license on their website, providing a clear description of their governance procedures, making sure that help materials are easy to find. The SBP committee strives at all times to reach consensus among the members, the provider and the broader community. As in any human endeavor, conflicts may arise when seeking to balance the interests of all parties. The committee therefore felt it important to document formal procedures for dealing with any issues that might arise (Standards and Best Practices Committee 2019b).

### Competing Standards and Best Practices

The SBP process was initiated to help those who need to use SBP’s in neuroscience to navigate the current options and to promote interoperability among neuroscience tools. One issue that must be addressed carefully is the issue of competing standards. Competing SBP’s should ideally be identified during the review process, either by the submitter, the review committee, or during the period of community comment. When competing SBP’s are identified, the committee determines whether having competing standards in a domain will be a significant impediment to further progress or if the field can support multiple standards without negative consequences. For example, during the reviews of PyNN and NeuroML, both standards for sharing computational models, the committee deemed that the field could support multiple standards without negative consequences; so they are viewed as complementary rather than competing, in that they are optimized for different conditions(Gleeson and Davison 2020). During the review of NWB:N 2.0, a standard for neurophysiology data, the committee determined that it overlapped with other standards for neurophysiology data, NIX and BIDS:EEG, and recommended that groups form an INCF working group so that they remain up to date on each groups’ efforts and work towards interoperability. When the committee determines that having competing standards constitutes a significant impediment to further progress in the field, the committee will invite the maintainers of the competing standards form a working group through INCF to work towards harmonization of the competing standards.

### Evolution of Evaluation Criteria

We expect that our understanding of what constitutes an effective standard will evolve as neuroscience continues to move towards collaborative, open, and FAIR-neuroscience. Indeed, there is an active effort in many domains to develop metrics for how to interpret FAIR (e.g., (Mons et al. 2017). Therefore, the SBP criteria themselves should have a clearly documented and community-based process for extension and updates.

The criteria listed in Table 2 were used for the reviews completed and underway (Table 3). However, not surprisingly, during the preparation of this manuscript, omissions were noted and modifications suggested. For example, Version 1 of the review criteria did not explicitly include extensibility as a criterion. What happens when new data types, hardware, tool, technology, or use case are introduced, as neuroscience evolves? It is common practice, given the diverse use cases and experimental landscape of neuroscience, to take an existing standard and extend or modify it for other use cases. BIDS, for example, has over 23 proposals for creating extensions to the core specification. The INCF and the SBP process are in a good position to provide a community-wide platform for discussions and consensus building about when a new standard is necessary vs extending an existing one.

### How Does the SBP Endorsement Process Help Neuroscience?

Why should an individual neuroscientist care? The adoption of clear and robust standards should also lead to a dramatic increase in the number, quality, interoperability and sustainability of tools and infrastructures. Our current model of funding tools and infrastructures through research grants leads to a lot of innovative ideas, but often less than useful or incomplete implementations. They advance the field of neuroinformatics, but they don’t always deliver working tools into the hands of the researcher that can propel discovery science. When a well defined standard becomes widely accepted, it provides the necessary uniformity and stability to reduce the overhead of tool development and to promote interoperability among tools so that researchers have a more powerful tool arsenal at their disposal. For example, well defined API’s can pass metadata and data between tools to avoid extra steps and so that provenance is maintained. A simple example is using ORCIDs for account management. As neuroscience adopts ORCIDs, users should be able to log into a resource like a data repository with their ORCIDs. The repository can automatically extract required details, e.g., affiliations, emails, from the ORCID database. At the same time, the repository can push information about data sets deposited by that researcher into their ORCID profile, much as ORCID is currently linked to databases such as PubMed.

On the data side, we often hear that “Data is the new oil”. But the extended metaphor goes on to state that “It’s valuable, but if unrefined it cannot really be used.” (Rotella 2012). Operationalizing FAIR for neuroscience is one of the key ways to ensure that data produced by the neuroscience community can be put to work, and community standards are essential for FAIR. While it is too early to measure the impact of the INCF endorsement process on community adoption, standards developed by the INCF network are having an impact on data quality and interoperability. For example, BIDS, the first standard endorsed by INCF, has a community of 136 credited contributors (22 female, as of October 3, 2020), with ~10,000 users visiting the website, and ~ 7000 users exploring the BIDS Specification, over the past 6 months. Over 404 journal articles have cited BIDS or any of its extensions. Currently, 10 reported centers, institutes and databases around the world that have implemented BIDS as their organizational structure. Furthermore, INCF has served as a convener of the standards developers and the large-scale brain initiatives which has resulted in harmonization/interoperability of the ontologies and metadata standards adopted by HBP and BRAIN Initiative infrastructure projects. More and more funders and journals are requiring that individual researchers publish their data so that it can be inspected and reused. We are starting to see good examples where pooling of smaller data sets leads to better powered studies and more reliable results (Ferguson et al. 2013; Lefebvre et al. 2015). Such studies suggest that publishing FAIR data will be of equal importance to publishing articles about findings derived from these data.

Today, INCF is well positioned to assume the role of a standards organization for neuroscience. Originally formed in 2005 to help neuroscientists to coordinate data and computational activities across international borders, INCF facilitated global cooperation for brain science in the very early days of neuroinformatics. The landscape has changed dramatically, as has the push towards open and FAIR neuroscience with INCF actively internalizing and adapting to those changes. As such, INCF has implemented a model for community standards development and adoption that empowers the broader neuroscience community to develop, evaluate, and endorse standards. Three important policies have been implemented to accomplish these goals: 1. SBP’s need not have been developed by INCF working groups to be considered, 2. the endorsement process includes community feedback, and 3. INCF does not just list SBP’s but actively evaluates them and works with standards providers to improve them when possible. The endorsement process is part of INCF’s strategy to develop a FAIR roadmap for neuroscience that provides researchers, infrastructure providers, tool developers, publishers, and funders with practical solutions for implementing the FAIR Principles in neuroscience. In addition to the endorsement process, the strategy also includes: 1. a portfolio of INCF endorsed SBPs that provides guidance on the appropriate use, implementation, and links to tutorials and tools/infrastructure that have implemented the SBPs, 2. Training and dissemination activities to promote community adoption, 3. a framework to identify areas in need of standardization, and 4. a framework for developing, extending, and harmonizing existing community standards.

Thus, INCF can serve as a neutral broker and coordination center on behalf of the wider neuroscience community to help coordinate and disseminate SBPs relevant for neuroscience. An INCF endorsement seal means that researchers, project managers, developers and funders can be confident in their choices. The community building experience and expertise with identifying and evaluating standards available in the INCF network also provides important expertise for those who are new to the practices of collaborative, open and FAIR neuroscience. As the process becomes better established, INCF can also provide a conduit for neuroscience-specific specifications to make their way into national and international standards organizations, to promote deployment in instruments and other commercial products supporting science. The training component of INCF will increasingly engage in training the communities to the use of the endorsed standards.

We encourage the neuroscience community to utilize the INCF network and expertise in identifying and evaluating additional standards, and to actively participate in this process through proposing SBP’s, providing feedback and joining or initiating INCF special interest groups (visit: https://www.incf.org/). As the amount of neuroscience data continues to grow, knowing how to make them open, FAIR and citable is an important skill and requirement to propel neuroscientific discovery in the twenty-first century.

## Figures and Tables

**Table 1:** A partial list of standards developed by INCF Task Forces or with INCF support. Active/inactive designations indicate whether the code base is being actively developed as of the writing of this manuscript

Standard/Best Practices	Description	INCF contribution	Available from:	Status

WaxholmSpace Mouse Atlas	A coordinate-based reference space for the mapping and registration of neuroanatomical data in the mouse brain.	Task Force	NITRC	Active
WaxholmSpace Rat Atlas	An open access volumetric atlas based on high resolution MRI and DTI, with Waxholm Space and stereotaxic space defined, shared in ITK-SNAP and MBAT- ready formats.	Task Force	NITRC	Active
Brain Imaging Data Structure	A standard for organizing neuroimaging and behavioral data	Meeting support	BIDS	Active
Neurodata Without Borders	A unified, extensible, open-source data format for cellular-based neurophysiology data	Task Force (initial work), Meeting support	NWB.org	Active
NIX	A data model and file format to store annotated scientific datasets	Task Force	GitHub	Active
Neuroimaging Data Model	A collection of specification documents and examples that describe an extension to the W3C PROV standard for the domain of human brain mapping.	Task Force	NIDM-NIDASH.org	Active
NineML	A simulator independent language for unambiguous description of spiking neuronal network models that aims to facilitate model sharing, portability, and re-usability.	Task Force	GitHub	Somewhat active; also SpineML, a community-led extension of NineML, is active
NeuroML	An XML-based description language that provides a common data format for defining and exchanging descriptions of neuronal cell and network models.	Support	neuroml.org	Active
Computational Neuroscience Ontology	A controlled vocabulary of terms used in Computational Neurosciences to describe models of the nervous system.	Task Force	BioPortal	Not active
Ontology for Experimental Neurophysiology	A controlled vocabulary of terms used to describe neurophysiology experiments	Task Force	GitHub	Not active
Common Mammalian Upper Brain Ontology	A reference framework for classifying general mammal nervous system structures.	Task Force	Terms available from InterLex	Not active

**Table 2:** Version 1.0 of the INCF endorsement criteria. These criteria were used to evaluate the SBPs indicated in Table 3. For the FAIR criteria, the relevant FAIR principle for each question is provided in the parentheses

Area	Criteria

1: Open	1.1 Is the SBP covered under an open license so that it is free to implement and reuse by all interested parties (including commercial)? 1.2 What license is used? 1.3 Does the SBP follow open development practices? 1.4 Where and how are the code/documents managed?
2: FAIR	2.1 SBP uses/permits persistent identifiers where appropriate (F1) 2.2 SBP allows addition of rich metadata to research objects (F2) 2.3 SBP uses/permits addition of appropriate PIDs to metadata (F3) 2.4 The protocol allows for an authentication and authorization when required (A1.2) 2.5 SBP uses or allows the use of vocabularies that follow the FAIR principles (I2) 2.6 SBP includes/allows qualified links to other identifiers (I3) 2.7 Does the standard interoperate with other relevant standards in the same domain? (I) 2.8 Does the SBP provide citation metadata so its use can be documented and tracked? (R1.2)
3: Testing and implementation	3.1 Does the SBP have a reference implementation? 3.2 What tools are available for the SBP? 3.3 Are the tools and implementations covered under an open source license? 3.4 What is your assessment of the quality of the code/document?
4: Governance	4.1 Does the SBP have a clear description of how decisions regarding its development are made? 4.2 Is the governing model document for maintenance and updates compatible with the INCF project governing model document and the open standards principles? 4.3 Is the SBP actively supported by the community? If so, what is the evidence? 4.4 Does the SBP provide tools for community feedback and support?
5: Adoption and use	5.1 Is there evidence of community use beyond the group that developed the SBP? 5.2 Please provide some concrete examples of use, e.g., publications where the use of the SBP is cited; databases or other projects that have adopted the SBP 5.3 Is there evidence of international use?
6: Stability and support	6.1 Does the SBP have a clear description of who is maintaining the SBP and 6.2 How is it currently supported? 6.3 What is the plan for long term support? 6.4 Are training and other supporting materials available?
7: Comparison	7.1 Are there other similar SBP’s available? 7.2 If yes, how do they compare on key INCF criteria?

https://space.incf.Org/index.php/s/Ypig2tfHOU4no8C#pdfviewer

**Table 3:** SBP’s that have been submitted for consideration for INCF endorsement and their status as of 12/19/2020

Standard or Best Practice Description	Date Nominated and by whom	Endorsement Status	Similar Standards

**Neurodata without Borders: Neurophysiology (NWB:N).** A unified, extensible, open-source data format for cellular-based neurophysiology data	3/8/2018 by Ben Dichter	Endorsed (Martone et al. 2020a) on 4/3/2020	NIX/odML BIDS EEG extension
**The FAIR Data Principles.** A set of guiding principles to make data and metadata Findable, Accessible, Interoperable, and Reusable	3/8/2018 by Jeffrey Grethe	In pipeline	
**NeuroML.** An XML-based description language that provides a common data format for defining and exchanging descriptions of neuronal cell and network models.	3/20/2018 by Padraig Gleeson	Endorsed (Martone et al. 2019b) on 3/20/2019	PyNN NineML SpineML
**Brain Imaging Data Structure (BIDS).** A standard for organizing neuroimaging and behavioral data	4/15/2018 by Chris Gorgolewski	Endorsed (Martone et al. 2018) on 11/1/2018	OpenfMRI schema NIDM Experiment EEG Study Schema XCEDE
**NeuroImaging Data Model (NIDM)-Results.** A standard that provides a representation of mass univariate neuroimaging analysis results, unified across analysis software packages	4/17/2018 by Camille Maumet	Identified as a candidate standard, but not ready for endorsement after community review on 11/9/2020	An extension of BIDS currently underdevelopment
**PyNN.** A simulator-independent language for building neuronal network models	4/17/2018 by Andrew Davison	Endorsed (Martone et al. 2019b) on 3/20/2019	NeuroML SpineML NineML
**Neo.** Python objects for neurophysiology data that could serve as a common object model for neurophysiology.	4/17/2018 by Andrew Davison	In progress	SpikeInterface NiBabel
**open metadata mark-up language (odML).** A standard metadata format for data annotation in electrophysiology	4/17/2018 by Thomas Wachtler	In progress	BIDS-EEG
**Neuroscience information Exchange (NIX).** A data model and file format to store annotated scientific datasets	4/17/2018 by Thomas Wachtler	Endorsed (Martone et al. 2020b) on 11/9/2020	NEO NWB:N NSDF (Neuroscience Simulation Data Format)

## Footnotes

1
https://www.internationalbraininitiative.org/sites/default/files/declaration-of-intent-september-2018.pdf

2
https://www.hl7.org/fhir/summary.html

3
https://www.commondataelements.ninds.nih.gov/

4
www.center-tbi.eu/

5
https://goo.gl/images/KaYDbJ

6
https://fairsharing.org

7
http://neuralensemble.org/neo/

8
http://co.mbine.org/

9
https://www.incf.org/about-us/history/incf-scientific-workshops

10
https://opendefinition.org/od/2.1/en/
